# Supplementary figures and images for: Frequent sugar feeding behavior by Aedes aegypti in Bamako, Mali makes them ideal candidates for control with attractive toxic sugar baits (ATSB)
Source: PLoS One. 2019 Jun 17;14(6):e0214170. doi: 10.1371/journal.pone.0214170 (PMC6576782; doi:10.1371/journal.pone.0214170)

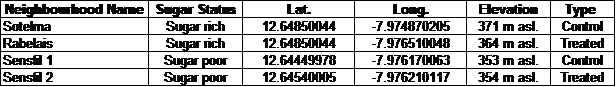

Supplement: S1 Table — (TIF) [file pone.0214170.s001.tif]
